# A Case of Poisoning and Recovery from the Use of Veratrum Viride

**Published:** 1860-05

**Authors:** N. O. Pearson

**Affiliations:** Palestine, Ill.


### A CASE OF POISONING AND RECOVERY FROM THE USE OF VERATRUM VIRIDE.

By N. O. PEARSON, M. D., Palestine, Ill.

As the Veratrum Viride is attracting considerable notoriety in the profession as a therapeutical agent, I shall give the history of a case of poisoning from its use. Mr. G. I., aged 35, of the nervous bilious temperament, and good constitution, was attacked Aug. 27th, 1858. Called to see him two hours from onset of disease.

*Diagnosis.*—He presented many of the symptoms of an ordinary acute attack of bilious fever—in fact I regarded it as such.

*Treatment*—I first administered a tea-spoonful of the following:

℞ Tinct. Veratrum Viride, ʒ ii.

Spts. Nit. Dulc. }

Tinct. Opii Camph. } a.a. ℥ i.

Mix. To be given every two hours until fever partially subsided, subsequently every four hours until entirely arrested.

But by mistake on the part of the nurse, it was given every half hour, until three doses were taken.

I was called again to see him six hours from the commencement of treatment, and found him presenting the following symptoms: Great prostration, with continual retching and vomiting; matters vomited white mucous; pulse 24 per minute, full and intermitting every sixth or seventh beat, indicating that the action of the heart was interfered with; respiration slow and laboring; body bathed in a cold perspiration. The cerebral functions were but little disturbed—the mind being clear. He remained in this condition six hours, at the end of which time reaction began to be established.

*Treatment during Paroxysm.*—Large doses of the emulsion of Carb. Ammonia, Opium, and its preparations, were alternately resorted to, together with stimulating applications and frictions, sinapisms of mustard, and hot rocks to the extremities.

*Remarks.*—Knowing that opium and its preparations are claimed as being antidotes in these cases, I availed myself of their administration in the most prodigal manner. How far they acted in correcting the poisoned condition of the system I cannot say. This case was undoubtedly caused by poisoning by veratrum viride, and should teach us to be very cautious in the use of so powerful a remedy. My direction in giving it as a sedative is this: to continue its use every one, two, or three hours, until it produces a livid hue of the cheeks; afterwards gradually diminish or entirely suspend it.

One thing worthy of note is this, that notwithstanding the amount of opium used, the patient had a free bilious evacuation from the bowels, and recovered without any other treatment whatever. Whether nature cured the disease, or the veratrum viride cut it short, I will leave the profession to judge.

